# The Journey Towards Withdrawal: Nurses’ Experiences of End‐of‐Life Decisions in Cardiothoracic Intensive Care

**DOI:** 10.1155/nrp/4969285

**Published:** 2026-09-22

**Authors:** Christina Björklund Pal, Katarina Sjögren Forss

**Affiliations:** ^1^ Department of Care Science, Faculty of Health and Society, Malmö University, Malmö, Sweden, mah.se

**Keywords:** cardiothoracic intensive care, end-of-life decision-making, ethical challenges, ICU nurses, interprofessional collaboration, qualitative research

## Abstract

**Background:**

End‐of‐life (EOL) decision‐making in intensive care units (ICUs) is ethically demanding. Nurses are often excluded from this process despite their continuous bedside presence. Surgical units, particularly cardiothoracic ICUs, are described as more inclined to pursue aggressive treatment, yet little is known about how nurses in these specialised settings experience EOL decision‐making.

**Aim:**

The study aimed to illuminate how nurses in the cardiothoracic ICU experience EOL decision‐making and the withdrawal of intensive care.

**Methods:**

A qualitative descriptive design was used. Twelve nurses from six cardiothoracic ICUs in Sweden were recruited through purposive sampling. Data were collected through digital semistructured interviews. An inductive content analysis following Elo and Kyngäs guided the analytic process.

**Results:**

Four categories captured the nurses’ experiences. Decision‐making as a winding road described the diverse and challenging nature of the decision‐making process. Endeavouring to be a part of the team reflected the importance of teamwork and the nurses’ inconsistent inclusion in EOL discussions. Facing ethical quandaries: Navigating doing good and doing no harm highlighted the ethical tensions inherent in intensive care. Caring beyond the patient: The crucial role of supporting relatives underscored the difficulties faced by relatives and the nurses’ commitment to caring for them.

**Conclusion:**

Cardiothoracic ICU nurses navigate significant ethical challenges during EOL decision‐making and emphasise the need for greater inclusion to strengthen advocacy. While several findings align with research from general ICUs, this study provides a context‐specific account of how EOL decision‐making is navigated in cardiothoracic intensive care. Persistent challenges related to nurses’ involvement and interprofessional communication highlight the need for strategies to support nurse inclusion in EOL decision‐making, as nurses’ contributions may strengthen patient advocacy and care for relatives during EOL transitions.

## 1. Introduction

Intensive care provides life‐sustaining treatment (LST) in the face of life‐threatening illness, compensating for failing vital functions [1]. When the underlying condition is irreversible, the appropriateness of continuing LST must be evaluated. Decisions to withhold or withdraw LST often precede the patient’s death, making end‐of‐life (EOL) decision‐making in the intensive care unit (ICU) particularly complex and ethically demanding [2]. ICU nurses characterise EOL decisions as a core ethical challenge, describing their work as living in constant ethical dilemmas between saving lives and accepting a natural death [3–5].

While physicians hold the formal responsibility for EOL decisions, ICU nurses seek to be involved, drawing on their continuous presence at the patients’ bedside and their role as advocates for patients and families [6, 7]. Nurses are often the first to recognise patients’ clinical deterioration, question the level of care provided and identify when a transition toward EOL care may be needed [8–11]. Nevertheless, they frequently report being excluded from decision‐making processes [11–13], which can leave them feeling unable to advocate effectively for their patients [14–18].

Notably, some studies suggest that EOL decision‐making perspectives may differ across ICU types. Surgical ICUs have been described as more inclined to pursue aggressive treatment and to delay transitions toward EOL care, compared to medical ICUs [15, 19]. However, it remains unclear how nurses in highly specialised surgical settings, such as cardiothoracic ICUs (CTICUs), experience EOL decision‐making and the withdrawal of intensive care. Despite the highly technological and intervention‐driven nature of cardiothoracic intensive care, which can complicate transitions toward EOL care, the literature has primarily focused on general or medical ICUs. Consequently, the unique characteristics of CTICUs remain unexamined, and research exploring CTICU nurses’ perspectives is scarce; only one study addressing this topic has been identified [20]. This limits understanding of how nurses navigate EOL decision‐making and withdrawal processes in this specialised context.

Given the ethical weight of EOL decisions, the specialised nature of cardiothoracic care and the limited available evidence, further exploration of CTICU nurses’ experiences is warranted. Therefore, this study aimed to illuminate the experiences of cardiothoracic intensive care nurses regarding EOL decision‐making and the withdrawal of intensive care.

## 2. Methods

This study employed a qualitative descriptive design [21]. Data were collected through semistructured individual interviews and analysed using inductive qualitative content analysis, as described by Elo and Kyngäs [22].

### 2.1. Context and Setting

There are eight CTICUs in Sweden, providing postoperative intensive care for patients undergoing cardiac, thoracic or vascular surgery. These units also offer extracorporeal membrane oxygenation and, in some cases, post–cardiac arrest care. A few units additionally manage heart and lung transplantation. CTICU nurses are registered nurses with a 1‐year master’s degree in intensive care, and they typically care for one or two patients at a time.

### 2.2. Sample and Recruitment

In qualitative research, sampling is guided by participants’ direct experience of the phenomenon under study [21]. Consequently, this study included CTICU nurses with documented experiences of EOL situations.

The study applied a purposive sampling strategy, selecting participants with relevant knowledge and experience of the research topic to ensure a rich contribution to the study [21]. In September 2024, an information letter was distributed to seven of the eight CTICUs in Sweden (one unit was excluded because one of the authors was employed there at the time). The letter was disseminated through two national networks (the Network for CTICU Nurses in Sweden and Healthcare Professionals in Cardiology) as well as through unit managers, who were asked to forward the information to all registered nurses employed at their units. Three reminders were sent out. Nurses interested in participating either contacted the first author directly via email or provided their contact details through the unit managers or network representatives. The first author subsequently contacted each nurse to arrange an interview. Twelve CTICU nurses participated in the study, representing six of the eight CTICUs in Sweden. All participants were employed at a CTICU at the time of the data collection. Their CTICU experience ranged from 2.5 to 27 years (a mean of 9.2 years), and their overall intensive care experience ranged from 3 to 38 years (a mean of 11.6 years).

### 2.3. Data Collection

Data were collected through semistructured individual interviews guided by open‐ended questions (Appendix 1), ensuring the coverage of key topics [21]. Probing questions were used to encourage participants to elaborate. Three pilot interviews with CTICU nurses were conducted to test the interview guide; the guide was subsequently revised, and one question was removed as it did not yield relevant data. The pilot interviews were not included in the final dataset.

The interviews were held between October and December 2024. All interviews were conducted digitally via Zoom, with the exception of one interview, which was conducted by telephone due to technical difficulties and the participant’s wishes. Two participants declined to have their cameras turned on during the interview. The interviews were recorded digitally and varied in length from 32 to 67 minutes (a mean of 46 minutes). They were transcribed verbatim before the analysis.

### 2.4. Data Analysis

The data were analysed using an inductive qualitative content analysis as described by Elo and Kyngäs [22]. An inductive approach is appropriate when the subject has not been thoroughly explored [22], making it suitable for this study. The analysis focused primarily on manifest content, while abstraction was applied during the development of categories.

First, the authors read the material several times to become immersed in the data. Codes describing the content were written in the margins. The codes were then collected in coding sheets, and similar codes were grouped together to form categories illustrating the nurses’ experiences. Finally, the data were abstracted by merging similar categories and sorting them under main categories (Table 1). The authors had continuous discussions during the analysis to enhance credibility, returning repeatedly to the raw data and making iterative adjustments until the analysis was completed [22].

**TABLE 1:** An example of the analysis process.

Interview excerpt	Open coding	Preliminary category	Category	Main category
“…being in those situations and being able to, at the same time, you want to convey hope but not too much hope either.” (ICU nurse K)	Wanting to give hope but not too much hope	Considering how one talks to relatives	Considering communication	Caring beyond the patient: The crucial role of supporting relatives

### 2.5. Ethical Considerations

In Sweden, ethical approval requirements are regulated by the Ethical Review Act [23]. This study, conducted within a Master of Science in Nursing programme, involved healthcare professionals only and did not include sensitive personal data or other elements requiring formal ethical review under the legislation. Therefore, formal ethical approval was not required. However, as ethical review requirements vary internationally, particular attention was paid to ensuring that the study adhered to internationally recognised ethical standards. Advice on ethical considerations was obtained from the Ethics Council at Malmö University. It should be noted that the Council serves an advisory function and does not provide formal ethical approval. The study was conducted in accordance with the Declaration of Helsinki [24], particularly its core principles of respect for individuals, informed consent, privacy and confidentiality. These principles guided all stages of the research process. All participants received written and verbal information about the study’s purpose, data handling and potential publication. Participation was voluntary, and participants were informed of their right to withdraw at any time without consequences. Oral informed consent was obtained prior to each interview. Confidentiality was ensured by coding transcripts and anonymising all quotations. No information that could directly identify participants or their workplaces was reported. Data were handled securely: Physical materials were stored in locked cabinets, and digital data were stored on password‐protected devices, accessible only to the authors, in accordance with applicable data protection regulations.

## 3. Results

**FIGURE 1:**
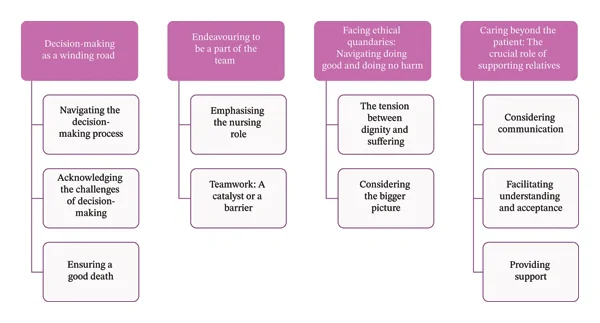
A structural overview of main categories and categories.

The analysis revealed four main categories, describing how the CTICU nurses experienced EOL decisions (Figure 1, Box 1). Throughout the results, “relatives” refers to the person(s) closest to the patient, and “team” refers to CTICU physicians, nurses and nursing assistants.

**Box 1: Core meanings of the four main categories.**
• Decision‐making as a winding road.
  Nurses described end‐of‐life decision‐making as a nonlinear process that often began with full escalation of care and gradually shifted toward reconsidering goals when deterioration persisted. They highlighted the challenges of delays and uncertainty in reaching withdrawal decisions and emphasised the importance of planning withdrawal to enable a dignified death when withdrawal became appropriate.
• Endeavouring to be a part of the team.
  Nurses portrayed the team as generally collaborative and supportive but described variation in how clearly their role was recognised and how consistently they were included in decision‐making. Team dynamics could function as either a facilitator or a barrier—shaped by communication climate, perceived physician authority and tensions between specialities that influenced the pace and direction of EOL decisions.
• Facing ethical quandaries: Navigating doing good and doing no harm.
  Nurses described intensive care as both enabling and ethically challenging, particularly when continued life‐sustaining treatment was perceived to prolong suffering without meaningful benefit. Ethical reflection extended beyond the bedside to considerations of quality of life after ICU and the wider implications of resource use when prolonged treatment limited capacity for other patients.
• Caring beyond the patient: The crucial role of supporting relatives.
  Supporting relatives was depicted as central, with nurses often acting as the continuity after physician conversations by clarifying information, preparing families for withdrawal and providing emotional support. Nurses described balancing honesty with hope and tailoring communication to relatives’ readiness, while striving to accommodate requests and protect time and dignity during the withdrawal process.

### 3.1. Decision‐Making as a Winding Road

The nurses described EOL decision‐making as a complex process. While they did not perceive EOL decisions as unusual, they stated that it was more common that patients died suddenly from acute complications, thus never getting to a situation of withdrawing.

#### 3.1.1. Navigating the Decision‐Making Process

The nurses described initially focusing on doing everything possible to save the patients. While remembering success stories and patients who had proved them wrong, they would often realise the probable outcome after a few days and see the need to discuss the direction of care in the team.

We start to notice it’s been a few days […] which way are we headed? Because it’s often a defining way to go. (ICU nurse K)

These discussions typically occurred during rounds, while the team considered options for moving forward with the care. The nurses approached the subject by asking questions regarding plans, prognosis and the physicians’ thoughts. Some nurses also described asking for potential limits to treatment. This aspect was rarely discussed, and other nurses explained that it could feel strange to limit treatment in an ICU.

You should be able to talk about not just, like, which other examinations can we do? What other operation can we do but what, what perhaps shouldn’t we do? (ICU nurse C)

According to the nurses, withdrawing LST was considered if the patient did not show any improvement or was deteriorating despite all available treatment. Patients’ wishes regarding LST were also considered. After rounds, the discussions then continued during interdisciplinary meetings, where physicians of other specialities could participate. Everyone in the team, including relatives, could raise the question of withdrawing LST. However, the nurses revealed that in some instances, no one did or the discussion lost momentum. The nurses described several situations where physicians pushed the question away and postponed decision‐making.

[Physicians should] not just push the [question of withdrawal] forward, because there is a tendency unfortunately to do that sometimes because you don’t feel comfortable or you think “Well, we’ll see.” (ICU nurse F)

#### 3.1.2. Acknowledging the Challenges of Decision‐Making

The nurses often perceived the decision‐making process as drawn out. Physicians took a long time to reach a decision, and the nurses waited without always knowing why. The nurses argued that waiting too long could result in a critical situation when nothing more could be done for the patient, thereby losing the chance of a dignified death. Therefore, treatment withdrawal should ideally be discussed in advance.

We who work closest to the patient […] often feel that the decisions are perhaps made too late, uhm, that we care and care for a patient that seems half dead. (ICU nurse B)

Nevertheless, the nurses understood the difficulties physicians face in finding the right time to make an EOL decision and the need for careful consideration. Taking time to decide was reasonable, and the nurses acknowledged that it was easy to say in hindsight that the decision could have been made earlier.

The challenge is that it gets indecisive sometimes, that we’re stuck considering, and it’s understandable that you have to consider because it’s such a hard decision that has to be made. (ICU nurse K)

The nurses identified the patients’ lack of improvement as a complicating factor in EOL decision‐making. This was especially true for surgical patients, where the surgery might have been successful but other organs failed. On the other hand, decisions were easier in irreversible situations, such as severe brain injuries or failure to wean off extracorporeal membrane oxygenation. Another factor complicating the decision‐making process was that the anaesthesiologist in charge rotated weekly; another physician could abruptly change the course of care. The nurses felt this led to uncertainty and stress for both them and the patients’ relatives.

Then you get a shock because you’ve really gone all in and just, yeah, investing completely and then the next person comes and just, “No, no, no, we’re not doing anything now.” (ICU nurse B)

#### 3.1.3. Ensuring a Good Death

Despite acknowledging withdrawal of LST as a part of their job, the nurses described it as emotionally demanding, especially if they were unable to provide a dignified end. The withdrawal could be a meaningful moment where they stood by their patient to the end.

It felt so important to try to provide this that we talk about, when we can’t alleviate or cure then at least a good death. (ICU nurse L)

Withdrawing LST could look different for each patient and was usually planned jointly by the team. During the planning, the nurses also raised the question of organ donation. Although the nurses opposed drawing out the withdrawal process, they also felt it sometimes happened too quickly after the decision was made. Occasionally, the nurses felt rushed by knowing other patients waited for available beds. The nurses expressed striving to provide a calm and dignified ending, promoting privacy and, in some instances, disregarding certain rules to accommodate both the patients and their relatives.

There were a lot of relatives, and the on‐call physician was first like, “OK, yes, we’ll bring in some of them” and then just, “No, we have to bring in more” and then like, sort of, “Fuck it. Everyone can come in.” (ICU nurse D)

### 3.2. Endeavouring to be a Part of the Team

The nurses described the team as working closely together around the patient. However, their own inclusion in EOL decision‐making was not always obvious.

#### 3.2.1. Emphasising the Nursing Role

Although interaction and communication within the team generally worked well, the nurses’ involvement in the decision‐making process varied. According to the nurses, their inclusion depended on the situation and ranged from participating in the EOL discussion to just being listeners. Some physicians discussed the decision with the team, while others only informed the nurses of the decision afterwards. Some nurses felt that these discussions were reserved for physicians, while others explained that they probably could participate but were not invited. Inclusion also comprised being informed and having a clear care plan. If such information was not offered, some nurses opined that they should be proactive and seek out the information they needed.

I want to participate so I can know what’s going on, what’s being planned. I mean, I have to know as much as possible so I too can provide good care and, above all, take care of the relatives in the best way as well. (ICU nurse L)

The nurses emphasised that they were not responsible for EOL decisions, and while they sometimes had differing opinions to physicians, they had a lot of respect for the physicians’ duty. However, they maintained that they should be able to express their views and contribute to the EOL decision‐making.

We often have a completely different idea of how patients are doing, how relatives are doing, and that’s why I think it’s much more important or much more rewarding in the process to involve us more frequently. (ICU nurse G)

The nurses’ role encompassed providing crucial information regarding the patients’ condition, ensuring that physicians had a solid basis for their decisions and that nothing was overlooked. Being closest to the patients, the nurses argued that they had a more comprehensive view of the situation, seeing beyond medical or surgical concerns. They strived to advocate for the patients and provide care of the highest quality regardless of the outcome.

[The nursing role] is very important because we sit by these patients’ bedside 24 hours a day […]. We have very good insight into like how the situation is, how fragile it is. (ICU nurse I)

#### 3.2.2. Teamwork: A Catalyst or a Barrier

According to the nurses, collaboration within the team was mostly well functioning. They described their colleagues as a valuable resource, offering support in tough situations. While they usually had informal discussions in the break room, several nurses reported also having structured debriefings after a withdrawal. This was deemed beneficial to process tough situations and facilitate learning and professional growth.

However, some nurses felt unsupported by the team, particularly by physicians. They described being met with harsh and condescending comments, which sometimes made them hesitant to speak up or raise the question of withdrawal. They also described their colleagues as being uncomfortable with the topic, perceiving it as taboo. Consequently, they saw a need for more open dialogue regarding EOL decisions. They wished for a more permissive environment, where talking about hard things was encouraged.

I’ve sometimes gotten the comment that “Yes but do you want the patient to die?” And that’s also like hard to hear that you’re perceived that way when you just want to raise a rather pertinent question during rounds. (ICU nurse B)

While patients in the CTICU were cared for by anaesthesiologists, the physicians responsible for their overall care were usually cardiothoracic surgeons, and decision‐making was a shared responsibility between the two. The nurses considered this a cause for issues in EOL decision‐making as they perceived surgeons to have opposing views regarding the withdrawal of LST.

It was rather obvious that the surgeons felt that there was a way forward anyway. But we who cared for the patient did not see it. (ICU nurse A)

Surgeons often wanted to continue treatment, delaying the decision‐making. The nurses speculated on whether this was related to statistics or prestige. They argued that surgeons had a fleeting presence in the CTICU and focused on surgical aspects, missing the bigger picture.

And [the surgeon] is the one who cut […]. It is his creation. (ICU nurse L)

### 3.3. Facing Ethical Quandaries: Navigating Doing Good and Doing No Harm

The nurses described intensive care as both a blessing and a curse in that sometimes too much could be done in terms of treatment, which opened for ethical issues.

#### 3.3.1. The Tension Between Dignity and Suffering

Although the nurses stated they could never know what the patients felt, they described intensive care as a painful experience. LST could lead to much suffering for both patients and relatives, and waiting for a decision could seem like an unnecessarily prolonged process.

You fight and fight. Why? When we see which way it’s going. You prolong the suffering for the patient, uh, you prolong the suffering for relatives. (ICU nurse E)

The nurses affirmed that they always considered whether providing LST was dignified and in the patient’s best interest. They questioned the use of LST in seemingly futile situations, describing it as unethical or wrong. For instance, they felt LST was sometimes taken too far, amounting to experimental treatments that did not resolve the underlying issues and making them strongly question why it was continued and whether it was worth it. These situations were described as especially challenging and causing feelings of distress and frustration.

You can always stick in another ECMO‐cannula, you can always (sigh) try some other variant of dialysis, you can always, yeah, do a bit of whatever to not withdraw. (ICU nurse C)

The nurses were driven by a wish to help the patient in any way possible. While burdened by losing patients, they simultaneously expressed an acceptance that patients sometimes died despite receiving all possible care. It was a part of life and should not be seen as a failure; it was impossible to save everyone. Although withdrawing LST was tragic, the nurses also described feeling relief once the decision had been made, especially if the process had been difficult.

#### 3.3.2. Considering the Bigger Picture

The nurses contemplated the quality of life the patient could have after intensive care. They acknowledged sometimes feeling that while patients perhaps could be saved, they would not return to a functional life. Such cases made them question the benefits and ethics of continuing LST.

Are [the patients] well mentally and physically or is it just suffering? What are we saving them to? (ICU nurse B)

Another perspective that the nurses brought up was one of society and cost. They argued that continuing LST for a patient who would not benefit from it limited the capacity of the CTICU. Consequently, taking too long to reach an EOL decision meant wasting resources, treating one patient at the expense of others.

Let’s say it’s a very intense treatment that’s extremely resource demanding […], then all the other patients […] may have to wait too long and deteriorate further because we don’t have the capacity to receive them in time. (ICU nurse G)

### 3.4. Caring beyond the Patient: The Crucial Role of Supporting Relatives

The nurses’ relationship with the patients’ relatives was depicted as a central one. The nurses stated that since patients usually were sedated or unconscious they would often feel closer to the relatives instead.

#### 3.4.1. Considering Communication

The nurses expressed carrying a large responsibility in the communication with relatives, making time to talk and being present during EOL conversations. Physicians often left after a conversation, whereas nurses stayed to offer further explanations and correct misunderstandings. In addition to reiterating medical information, the nurses explained everyday things that were done to care for the patient. They deemed involving relatives and helping them understand the situation essential.

The nurses described choosing their words carefully when speaking to relatives, balancing between giving them hope and remaining honest about the severity of the situation. They emphasised the importance of a clear and direct communication that left no room for misunderstandings, as well as not making any promises. They explained trying to gauge what the relatives knew and understood, not wanting to preempt a conversation with the physician or give the wrong impression. Thus, remaining neutral could be particularly challenging.

Us nurses, we give politician answers all the time. (ICU nurse H)

#### 3.4.2. Facilitating Understanding and Acceptance

According to the nurses, it was crucial to have relatives on board. There were cases when relatives could not process the situation or kept hoping for a different outcome. For instance, receiving contradictory information from physicians or not discussing LST withdrawal could hinder understanding, and relatives would then interpret ongoing treatment as hope. However, most relatives understood where the situation was headed if given enough time.

Many relatives have been there the whole time and seen how we work to save the patient and understand that it won’t work. (ICU nurse J)

Relatives sometimes had fearful preconceptions about withdrawing LST, and the nurses strived to prepare them, for example, by explaining possible scenarios. When relatives needed more time to understand and accept the situation, the nurses thought it acceptable to delay withdrawal if possible.

The nurses saw relatives having varying reactions to an EOL decision. While most were sad but accepting, some remained shocked or questioned whether more could have been done. The decision to withdraw LST could in some instances bring a sense of relief, but sudden illness or young age encompassed a more traumatic experience.

#### 3.4.3. Providing Support

The nurses described relatives being caught between hope and despair. Witnessing their struggles and suffering, tossed between these extremes, was difficult for the nurses. Therefore, they strived to be present, offering support and comfort in different ways, or to bring in counsellors or spiritual leaders.

The nurses thought it paramount to give relatives as much time as possible with the patient. They also made a point of asking relatives about any requests for the withdrawal and did their utmost to accommodate such wishes.

The relatives wanted the patient to go outside the walls of the ward and just get to experience fresh air one last time, so then we brought the whole shebang with us and went up to the helicopter pad. (ICU nurse F)

Despite the tragic circumstances, the nurses described relatives generally being grateful and pleased with how the withdrawal was handled. Such reactions brought comfort for the nurses as well.

But we dressed a young girl in her graduation dress after she passed away. One of those small ones with little thin straps, very short, very tight, and both of us in the room thought how, how is this going to work? But it went well, she got it on and she looked really lovely. Her mother was very very pleased. (ICU nurse J)

## 4. Discussion

The findings show that CTICU nurses experience EOL decision‐making as a complex and ethically challenging process, where relationships with patients’ relatives, teamwork and inclusion are central concerns. While previous research has mainly addressed general ICU contexts, the experiences of CTICU nurses in this study suggest important continuities, while also highlighting how these issues unfold in a cardiothoracic setting: The weekly rotation of the physician in charge disrupts continuity [8, 11, 14, 25]; nurses possess a holistic view, advocate for patients and strive to ensure a dignified death [6, 9, 16, 17, 25]; and they wish to be included in the decision‐making process [10, 17]. Moreover, nurses consider supporting relatives essential in EOL situations, particularly after the decision to withdraw LST [26–28]. The CTICU nurses in this study emphasised careful communication with relatives, echoing statements of nurses in Calvin et al.’s study [20], who underscored the importance of guiding families toward acceptance without being overly direct. However, relatives may still perceive nurses’ communication as vague [29]. The CTICU nurses in this study expressed aiming to support relatives while simultaneously protecting them from additional distress, illustrating the emotional and moral complexity of EOL communication.

The nurses perceived cardiothoracic surgeons to have opposing views to the CTICU team during EOL decisions, which could hinder teamwork and delay withdrawal of LST. Other studies similarly describe surgeons’ reluctance to limit treatment or discuss prognosis [19, 30–32]; even anaesthesiologists have observed these issues in general intensive care [33]. In this study, the CTICU nurses considered delayed EOL decisions a cause of patient suffering and a contributor to nurses’ moral distress, a well‐documented phenomenon in previous research [7, 11, 14, 16, 18, 34]. The nurses in this study described receiving harsh comments from physicians when raising the question of withdrawal. Calvin et al. [20] also note this issue but do not specify whether the physicians are surgeons or anaesthesiologists. This raises the question of whether differences between medical and surgical ICUs are related to the surgical profession or stem from the CTICU itself. Regardless, these situations make nurses feel unheard and hinder teamwork, indicating an issue that warrants further attention.

Such tensions may also reflect differences in role‐based responsibilities and moral pressures, underscoring the importance of structured interprofessional dialogue around EOL decisions. Michalsen et al. [35] argue that interprofessional shared decision‐making in the ICU can support EOL decisions by facilitating information exchange, deliberation and joint understanding within the team. Recent Scandinavian consensus statements echo this by recommending inclusion of ICU nurses in multidisciplinary conferences that precede EOL decisions [36]. However, global evidence suggests that the conditions for such collaboration, as well as nurses’ involvement in EOL decisions, vary considerably [37]. This resonates with the present findings and suggests that improving both structure and climate may help address the teamwork barriers described in CTICU EOL decision‐making [35, 36].

As ICU patients are often unconscious or sedated, their autonomy is difficult to uphold. Consequently, the CTICU nurses in this study saw their role as patient advocates as crucial, and their proximity to both the patients and their relatives provided unique insights. This position allows nurses not only to bring a holistic perspective into EOL decision‐making but also to promote patient‐ and family‐centred care [26, 38]. Although they are not responsible for the final decision, nurses can contribute substantially to the process. This highlights both the value of their involvement and the complexity of their role within a medically dominated field [39]. Therefore, the benefits of involving nurses in EOL decision‐making should not be underestimated.

### 4.1. Methodological Considerations and Limitations

The study has a relatively small sample, which limits transferability. However, in qualitative research, sample size is not determined by numerical criteria but by the quality and relevance of the data in relation to the study aim [40]. These considerations align with the concept of information power, where samples with high specificity and rich data allow for smaller samples [41]. Thus, the modest sample size can also be understood as enabling in‐depth exploration of participants’ experiences, which is a hallmark of qualitative inquiry [42]. Furthermore, the inclusion of ICU nurses with heterogeneous professional backgrounds from multiple hospitals across the country provides contextual breadth and variation, strengthening the credibility of the findings despite the limited number of participants.

However, the recruitment process may have introduced selection bias due to the reliance on intermediaries for information dissemination; representatives and unit managers acted at their own discretion, potentially resulting in uneven access among eligible participants. Additionally, because participation was voluntary, the sample may reflect self‐selection bias, whereby individuals with stronger views or greater motivation to discuss the topic are more likely to participate [43]. This may have led to an overrepresentation of particularly striking experiences, while more neutral or routine perspectives may be less visible. While the nurses in this study described a range of experiences, both positive and challenging, self‐selection may nonetheless have shaped the range and emphasis of perspectives captured.

Conducting digital interviews via Zoom enabled a nationwide inclusion of CTICUs and participants and offered advantages such as flexibility and the ability to observe nonverbal cues [44]. Zoom is particularly suitable for qualitative interviews [45]. Conversely, the digital format may have influenced sampling by excluding individuals with limited access to or comfort with the technology [44]. One of the authors’ prior experiences in CTICU settings provided valuable contextual understanding but also posed a potential risk of bias. To mitigate this, continuous reflexivity and regular discussions with the co‐author were maintained throughout the study [46]. Trustworthiness was addressed at all stages of the research process through appropriate sampling, thorough documentation and adherence to SRQR guidelines for qualitative reporting [47]. Participant quotations were included in the results to illustrate the findings and strengthen credibility [46].

## 5. Conclusions

CTICU nurses navigate significant ethical challenges during the EOL decision‐making process while striving to care for both patients and relatives. They wish to be more involved in the decision‐making process, viewing inclusion as essential for patient advocacy and holistic care. Although several aspects of CTICU nurses’ experiences resonate with findings from general ICU settings, this study adds a context‐specific account of how EOL decision‐making and withdrawal of intensive care are navigated in a highly technological, surgical intensive care environment. The persistence of challenges related to nurses’ involvement and interprofessional communication underscores the need to move beyond further description toward practice development on how nurses’ contributions can be integrated more consistently.

Therefore, future research should examine how nurse inclusion in EOL decision‐making can be implemented, as nurses’ contributions may support patient advocacy and strengthen care for relatives during EOL transitions. Moreover, exploring the perspectives of both anaesthesiologists and cardiothoracic surgeons could further improve communication and teamwork during these processes in the CTICU.

## Funding

No funding was received for this manuscript.

## Conflicts of Interest

The authors declare no conflicts of interest.

## Supporting Information

Additional supporting information can be found online in the Supporting Information section.

## Supporting information


**Supporting Information** Appendix 1. Interview guide used during data collection is provided as a supporting file.

## Data Availability

The data that support the findings of this study are available on request from the corresponding author. The data are not publicly available due to privacy or ethical restrictions.
