# Supplementary material for: The Journey Towards Withdrawal: Nurses’ Experiences of End‐of‐Life Decisions in Cardiothoracic Intensive Care
Source: Nurs Res Pract. 2026 Sep 22;2026:4969285. doi: 10.1155/nrp/4969285 (PMC13595814; doi:10.1155/nrp/4969285)
Supplement: Supplementary file 1 [file NRP-2026-4969285-s001.docx]

**APPENDIX 1: INTERVIEW GUIDE**

**Background questions:**

What is your work experience within cardiothoracic intensive care? Any work experience within other intensive care contexts?

Who is the physician in charge (for example, intensivist, anaesthesiologist, cardiologist, cardiothoracic surgeon)?

Who carries the main responsibility for end-of-life decisions and withdrawal of life-sustaining treatments?

**Please tell me about your experiences regarding end-of-life decision-making and the withdrawal of life-sustaining treatment for patients in the CTICU.**

Examples of follow-up questions: What is it like for you as a CTICU nurse when LST is withdrawn? What does it mean for you to be a CTICU nurse in these situations?

**Based on your experiences, what does the end-of-life decision-making process look like?**

Examples of follow-up questions: What happens before the decision is made? What do the discussions entail? Who is involved and in what manner? What is your role and responsibility in the process? Can you tell me about the communication and teamwork in these situations?

**Examples of probes:**

Can you tell me more about…?

Can you give me an example of such a situation? A particularly positive or challenging situation?

What made that moment stand out?

What happened then?

What did you think when…?

Is there anything you’d like to add?

Is there anything about the topic that you’d want to highlight in particular?
